# Antagonistic and Quantitative Assessment of Indigenous Lactic acid Bacteria in Different Varieties of *Ogi* against Gastrointestinal Pathogens

**DOI:** 10.11604/pamj.2017.27.22.9707

**Published:** 2017-05-10

**Authors:** Ayorinde Oluwatobiloba Afolayan, Funmilola Abidemi Ayeni, Werner Ruppitsch

**Affiliations:** 1 Department of Pharmaceutical Microbiology, Faculty of Pharmacy, University of Ibadan, Ibadan, Oyo State, Nigeria; 2 Austrian Agency for Health and Food Safety, Institute of Medical Microbiology and Hygiene, Vienna, Austria

**Keywords:** Ogi, omidun, salmonella, coculture, fermentation, acetobacter

## Abstract

**Introduction:**

*Ogi* is a popular fermented cereal gruel consumed mainly in the western part of Nigeria. Traditionally, uncooked *Ogi* is normally administered to diarrhoea patients to reduce the frequency of stooling. This study was therefore undertaken to identify, quantify and determine the antimicrobial properties of lactic acid bacteria (LAB) isolated from Ogi.

**Methods:**

The *Ogi* samples (Yellow, white, sorghum) were obtained from different market in Ibadan, Nigeria and Ogi control (cooked, uncooked and Omidun) were prepared with the viable counts of bacteria monitored over 5 days period. LAB were isolated from the varieties and identified by partial sequencing of 16S rRNA gene. The antimicrobial activities of the cell free supernatant (CFS) and the viable cells of the isolated LAB against *Escherichia coli* EC004, *Salmonella* sp. SS11, *Shigella* sp. SS10 were investigated by agar diffusion assay, agar overlay method, and coculture growth studies.

**Results:**

*Omidun* had the highest LAB count while cooked ogi has the lowest LAB count. *Weissella paramesenteroides , L. brevis, L. rossiae, L. fermentum, L. plantarum, Acetobacter pasteurianus, Paenibacillus* sp. and *Bacillus* sp. were isolated from Ogi in this study. Large zone of inhibition (11≤x≤20) was observed with CFS against *Salmonella* sp. SS11 and *Shigella* sp. SS10 and also the overlay method. Coculture studies of *Weissella paramesenteroides, Lactobacillus fermentum*, and *L. plantarum* with *Salmonella* sp. SS11 showed a 5-8 log reduction of the pathogens' growth after 24 hours as compared with the control.

**Conclusion:**

*Ogi* and its contents have antimicrobial properties against pathogenic organisms.

## Introduction

The gastrointestinal microflora helps to maintain a microbial barrier against the colonization and proliferation of pathogens in the digestive tract [1]. Pathogens are responsible for inciting intestinal infections that negatively affect the normal functions of the gastrointestinal tract, leading to diseases such as cholera, typhoid, salmonellosis, acute gastroenteritis, traveller's diarrhoea and shigellosis. These aforementioned diseases are characterized by a common symptom known as diarrhoea. Diarrhoea is an illness characterized by stools in increased frequency and fluidity and it is one of the most common illness causing infant death in developing countries [2]. Consumption of contaminated food, poor hygiene, and close proximity to animals are the reasons why pathogens (such as *Escherichia coli, Salmonella* species, *Shigella* species, *Staphylococcus aureus, Clostridium difficile* and *Campylobacter jejuni*) find their way to the gastrointestinal tract [3]. Diarrhoea, although self-limiting, may sometimes require antibiotic therapy. However, most of the pathogens especially bacteria have already developed resistance to most of the conventional antibiotics [4]. Therefore it is necessary to look into scientific basis of some traditional remedy for diarrhea through the use of fermented foods that naturally contain beneficial microorganisms that will help to successfully compete with, and inhibit the growth of the gastrointestinal pathogens.

Ogi is an acid fermented cereal gruel made from maize or corn (*Zea mays*), sorghum (*Sorghum vulgare*,), and millet (*Pennisetum americanum*) [5]. It is the most popular traditional health-sustaining fermented food in Western Nigeria, and serves as weaning foods for infants in this region. Ogi consists of smooth cereal sediments and fermented water on top called Omidun. In some communities in southwestern Nigeria, raw ogi is normally administered to people suffering from gastroenteritis to reduce/minimize discomforts [6, 7]. Lactic acid bacteria have been associated with the fermentation of Ogi and have been frequently isolated [8]. The nutritional benefits of Ogi, microbial diversity and the roles of fermenters against inoculated pathogens in ogi have been investigated extensively [8, 9] but no information is available on antagonistic effects of LAB isolated from ogi against gastrointestinal pathogens in co culture. Furthermore, Akharaiyi et al [10] and Omemu and Omeike [11] investigated effect of heat on general flora in Ogi but no information exist on differing quantities of LAB in cooked, uncooked ogi and Omidun. Since probiotic effect of LAB is dependent on admistration in large number, such information could provide important data to establish the best form of Ogi with high quantities of LAB present with their associated health benefits. This study therefore aims to discover the diversity and quantities of LAB in different varieties of Ogi (White Ogi, Yellow Ogi, and Sorghum Ogi, the form of Ogi with highest quantities of LAB (cooked, uncooked, Omidun) and associated antimicrobial activities in co culture.

## Methods

**Collection of gastrointestinal pathogens**: Multidrug resistant gastrointestinal pathogens used in this study, Escherichia coli EC004, Salmonella sp. SS11 and Shigella spp SS10 were obtained from the culture collection of Ekiti State University Teaching Hospital, Ekiti State, Nigeria and OTA Catholic Hospital, Oluyoro, Ibadan, Oyo State, Nigeria. The isolates were 60% resistant to antibiotics.

**Enumeration and isolation of viable vactic acid bacteria in ogi samples**: Three varieties of traditionally prepared samples of fermented ogi [White maize (Zea mays),Yellow maize (Z. mays, yellow variety),and Red guinea corn (Sorghum vulgare)] were obtained randomly from four different markets in Ibadan, Oyo State, South-West, Nigeria. The Ogi control was prepared according to the method of Odunfa and Adeyele [12] with slight modifications. Two hundred grams of the white variety of the cereal grains was weighed into 300 ml sterile distilled water and steeped for 72 hours at 28 ± 2^o^C. The water was decanted and the grain was wet-milled using properly washed grinding machine. The resulting pastes were sieved using sterile muslin cloths, the filtrate collected into a sterile container and allowed to settle for 3 days during which fermentation took place by the natural flora of the grains. The LAB in Omidun (ogi supernatant), and the uncooked Ogi slurry (of each of the 5 fermentation days) were enumerated by standard procedures. Cooked Ogi was prepared by separately heating the slurry of the fermented ogi sample in boiling water under constant stirring using a clean stirrer to form a thick paste. The LAB in the cooked Ogi were enumerated by standard procedures. Lactic acid bacteria were isolated from the four different Ogi varieties by inoculating 1 gram of each Ogi variety into MRS broth (Oxoid, UK) and incubated microaerophilically for 24 hours. Afterwards, distinct colonies were picked at appropriate dilution. All pure cultures were stored in 50% MRS broth/ glycerol at -200C for subsequent studies. For the fermented Ogi Control, starting from the 1st fermentation day up to the 5th fermentation day, LAB were isolated from Omidun, Uncooked Ogi, and Cooked Ogi (starting from the 3rd day of fermentation).

**Identification of isolated lactic acid bacteria**: The DNA of bacterial isolates were extracted by QuickExtractTM DNA extraction solution (Epicentre, Wisconsin) according to the manufacturer´s instructions. PCR samples were prepared in a total volume of 20µl containing 1µl of DNA extract, 10 pmol of each primer, and 25 µl of 2-fold concentrated RedTaq Ready Mix (Sigma). The oligonucleotides used for amplification correspond to the 5' end (5'-TGTAAAACGGCCAGTAGAGTTTGATC(AC)TGGCTCAG) and the 3' end (5'- CAGGAAACAGCTATGACCG(AT)ATTACCGCGGC(GT)GCTG) containing an M13 primer sequence [13, 14] PCR conditions were 95°C for 5 min; 35 cycles each of 95°C for 15 s, 58°C for 30 s, and 72°C for 45 s; and a final step at 72°C for 10 min. Prior to sequencing, 10 µl of the amplified products were analyzed on 1.5% agarose gels and 5µl were purified with EXO SAP-IT (GE Healthcare, Buckinghamshire, GB). Two µl purified amplification product were used for subsequent sequencing with primers M13 universal (5´-TGTAAAACGACGGCCAGT-3`) and M13 reverse (5´-CAGGAAACAGCTATGACC-3`) (Eurofins MWG Operon, Ebersberg, Germany) using the BigDye Terminator v3.1 sequencing kit (Applied Biosystems, Carlsbad, California). DNA sequencing was performed as previously described [15]. Products were analyzed on an ABI Genetic Analyzer 3500Dx (Applied Biosystems) according to the manufacturer´s instructions. The obtained forward and reverse sequences of each sample were assembled and edited using the AlignIR software, version 1.2 (LI-COR). Each consensus sequence was blasted against the NCBI database for species identification. The sequences have been deposited in GenBank with accession numbers KU725800-KU725823.

**Determination of in vitro Antimicrobial Activity of the LAB Isolates against Pathogenic Bacteria**: (a) Viable Cells Overlay Assay This method has been described by Ayeni et al [16]. The LAB cells were innoculated in two 2-cm-long lines on a MRS agar surface and then incubated at 37 °C for 24-48 h in microaerophilic conditions. The plates are overlaid with 0.2 ml of an overnight broth culture of Escherichia coli EC004, Salmonella SS11 and Shigella spp SS10 inoculated in 10 ml of soft Nutrient agar (0.75% agar-agar; Oxoid, UK). The plates were incubated aerobically at 37 °C for 24 h. The inhibition activity is indicated by the clear zones around the line of the LAB. (b) Cell-free Supernatant Assay The antimicrobial activities of the cell-free culture supernatants of isolated LAB against Salmonella sp. SS11 and Shigella spp SS10 were determined by agar well diffusion assay. The diameters of the growth inhibition zones were recorded in millimetre (mm). (c) Coculture Assay The observed relative susceptibility of Salmonella sp. SS11 made the pathogen to be selected for coculture study. The interference of selected LAB with the growth of pathogenic strains was evaluated by coincubating gastrointestinal Salmonella spp SS11 with three representative strains of LAB {Weissella paramesenteroides AFN004, L. fermentum AFN018 and L. plantarum AFN021} according to the method of Drago et al [17]. This was done in two series of experiments. In the first experiment, overnight culture of Salmonella spp was inoculated into 5 ml double strength Nutrient broth and then added to the overnight culture of LAB and the mixture incubated for 24 hrs. The viable cells in the mixture were evaluated at t0 and t24h by plating out appropriate dilution on MRS agar and Salmonella-Shigella agar to evaluate surviving LAB and Salmonella spp. respectively. The monoculture of the LAB and gastrointestinal pathogens were also evaluated. For the second experiment,. Salmonella spp were inoculated into fresh Nutrient broth and incubated for 8 hrs, after which the pathogen cultures were centrifuged (for 10 min at 10 000 rpm), resuspended in 5ml double strength Nutrient broth and added to 5 ml of overnight culture of LAB. Plating of both the pathogen alone (monoculture) and the mixture (coculture) were done at 8 hrs and 24h on their respective media to enumerate viable cells.

## Results

**Figure 1:**
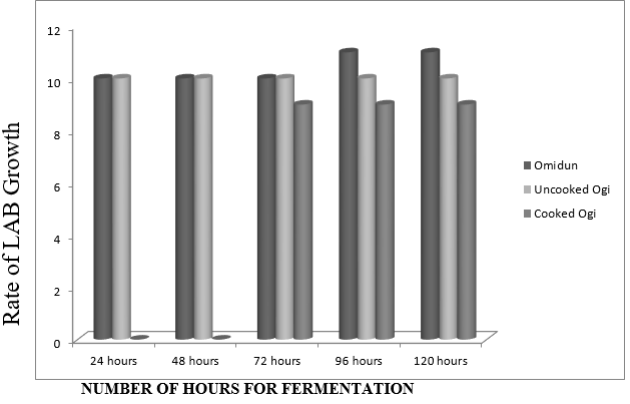
Microbial (Lactic Acid Bacteria) load of white ogi control. Y- axis represents the rate of LAB growth revealed as the exponent of 10 (exponents are within the range of 0 < y<12)

**Figure 2:**
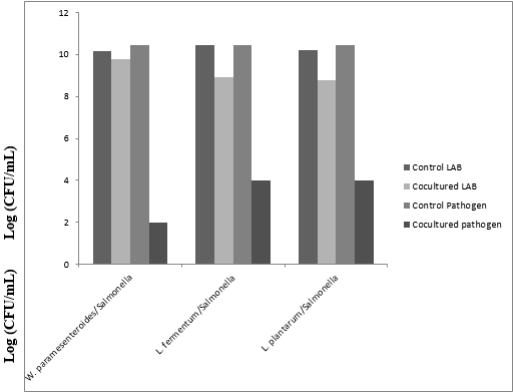
Inhibition of in vitro growth of salmonella sp. IBD 011 by W. paramesenteroides AFN004, L. fermentum AFN018, and L. plantarum AFN021 in coculture

**Figure 3:**
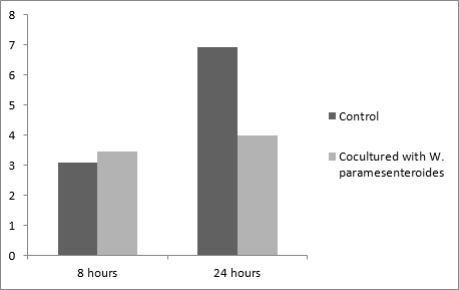
Inhibition of in vitro growth of salmonella by W. paramesenteroides AFN004 (8 hours and 24 hours)

Different samples of three varieties of Ogi (Yellow, Brown, White) were purchased from Ibadan metropolis in Western Nigeria The mean viable counts of LAB present in different Ogi samples reveals that Yellow Ogi has 4.8 × 1011 while Red Ogi has 3.8 × 1011 but the least count is from white ogi (2.0 × 1010) Also, in Ogi control, the viable counts of LAB in Omidun increases with increasing days of fermentation, from 2.0 × 1010 in Day 1 to 3.0 × 1011 in day 5. The uncooked Ogi maintained a steady count of 1010 cfu/ml throughout the 5 days of fermentation while cooked Ogi has the lowest count of 109 cfu/ml Figure 1. A total of 27 LAB strains were identified from the different varieties of Ogi and the Ogi control as L. plantarum (36%), L. fermentum (24%), Lactobacillus brevis (20%), Acetobacter pasteurianus (8%), Weissella paramesenteroides (8%) and L. rossiae (4%) Table 1. The antimicrobial activities of LAB isolates against the 3 gastrointestinal pathogens are shown in Table 1. 21 (75 %) LAB were active against Shigella sp. SS10, 19 (67.86 %) were active against Salmonella sp. SS11, 18 (57.5 %) were active against Escherichia coli EC004. The cell free supernatant from most of the isolates had antagonistic activity against the test pathogens. 19 (73.08 %) LAB was active against Shigella sp. SS10 and Salmonella sp. SS11. L. plantarum strains were generally active against the test pathogens Table 1. The capability of the lactic acid bacteria strains to inhibit Salmonella sp. SS11 growth was evaluated in coculture experiments, which were carried out in two parts. In the first series of experiments, W. paramesenteroides AFN004, L. fermentum AFN018, and L. plantarum AFN021 inhibited the growth of the pathogens Salmonella sp. SS11 with 6-8 log reduction. The highest log reduction was exhibited by W. paramesenteroides AFN004 (7 log reduction) Figure 2. In another experiment where Salmonella SS11 has grown for 8 hours before introducing a selected active LAB (W. paramesenteroides AFN004), the LAB inhibited the pathogen by 4 log in comparison to the control Figure 3.

**Table 1:** Characterization of lactic acid bacteria from different varieties of ogi

			CFCS(mm)		Viable Cells (mm)		
Ogi Samples (No of Samples)	LAB ISOLATED	CFU/ml (range)	*Salmonella* sp. SS11	*Shigella* sp. SS10	*E.coli* EC4	*Salmonella* sp. SS11	*Shigella* sp. SS10
Agbowo Yellow (1)	*L. fermentum* AFN012	4.0 x 10^11^ – 4.8 x 10^11^	16	16	20	20	20
	*A. pasteurianus* AFN024		0	0	0	22	23
Agbowo Brown (1)	L. brevis AFN011	2.7 x 10^11^ – 3.8 x 10^11^	18	16	0	0	0
	*L. plantarum* AFN036		15	16	0	18	18
Sango Yellow(1)	*L. plantarum* AFN032	4.0 x 10^11^ – 4.8 x 10^11^	15	16	18	20	21
Iwo Road Yellow (1)	*L. rossiae* AFN016		13	12	0	20	20
	*L. fermentum* AFN035		18	15	20	20	16
Iwo Road White (1)	*L. brevis* AFN005	1.6 x 10^10^– 2.0 x 10^10^	0	15	0	0	0
	*L. brevis* AFN030		0	0	0	0	0
	*L. plantarum* AFN039		0	14	0	20	18
Iwo Road Brown (1)	*L. fermentum* AFN018	2.7 x 10^11^ – 3.8 x 10^11^	0	12	15	0	20
	*W. paramesenteroides* AFN028		16	17	20	15	0
Ojoo Yellow (1)	*L. plantarum* AFN043		20	19	20	20	7
	*L. plantarum* AFN040		17	0	20	15	20
Ojoo Brown (1)	*L. fermentum* AFN045		18	17	20	20	15
Uncooked White Ogi (5)	*L. plantarum* AFN015 (D 5)	1.07 x 10^10^– 2.9 x 10^10^	0	0	20	20	20
	*L. plantarum* AFN021 (D 5)		19	19	20	15	21
	*L. plantarum* AFN033 (D 5)		13	15	20	20	20
	*W. paramesenteroides* AFN004 (D1)		15	18	20	0	20
Cooked White Ogi (3)	*A. pasteurianus* AFN014 (D 5)	1.84 x 10^8^– 6.6 x 10^9^	18	18	20	17	20
	*L. plantarum* AFN023 (D 5)		18	13	20	24	25
Omidun (5)	*L. brevis* AFN007 (D 5)	2.2 x 10^10^ – 3.2 x 10^11^	20	17	20	10	11
	*L. brevis* AFN029 (D 5)		0	0	20	20	20

Note: D is day

## Discussion

One of the properties of probiotics is presence of viable cells in adequate amount to confer health benefits. In a country like Nigeria that lacks approved probiotic formulation in its market, then the health benefits of fermented foods can be considered in relation to quantities and qualities of the fermenting LAB. In this study, it was observed that omidun (the ogi supernatant) had the highest load of LAB followed by the uncooked ogi slurry while cooked ogi has the lowest number of viable LAB. These observations also reinforce the standard knowledge that cooking reduces beneficial microorganisms in foods. Uncooked ogi is normally administered to persons suffering from diarrhoea-related infections in order to reduce stooling frequency [6], however, Ogi is usually eaten cooked. The traditional practice of cooking Ogi kills beneficial bacteria, thereby reducing the quantity of viable LAB present in this fermented staple food. In order to achieve the optimum potency resulting from the high quantity of LAB present, consumption of the uncooked Ogi rather than cooked Ogi should be encouraged as it is widely done in some Nigerian cultures. However, Omemu and Omeike [11] stated the likelihood of pathogenic contamination in improperly cooked Ogi, therefore consumption of raw Ogi should only be when the preparation is under clean conditions. as was done in this study. Weissella paramesenteroides , L. brevis, L. rossiae, L. fermentum, L. plantarum, Acetobacter pasteurianus, Paenibacillus sp. and Bacillus sp. were isolated from Ogi in this study. The commonest species is L. plantarum. In a study by Okeke et al [18], Pediococcus sp. dominated white and yellow ogi while Pediococcus sacidilactici and Lactobacillus paraplantarum were found during fermentation. Lactobacillus and Leuconostoc have been reported as the occurring genera of lactic acid bacteria (LAB) in Omidun and fermented /souring ogi Okeke et al [18], but no Leuconostoc sp was isolated in the present study.

The dominance of L. plantarum in other Nigerian fermented foods has been confirmed [19]. The higher prevalence of rod-shaped LAB in this study corroborated a previous study [20]. Also, Sanni et al [21] reported that L. plantarum and L. fermentum strains were also isolated at a high frequency of 24.6 and 26.3%, respectively during fermentation of Ogi. However, the presence of the species Lactobacillus rossiae and Weissella paramesenteroides in Ogi has not been previously reported. Previous studies have revealed that L. rossiae is a highly versatile species capable of colonizing different environments, such as fermented cereals, legumes, fruits and meat, as well as being an inhabitant of the human and animal gut-intestinal tract [22–24]. Its presence in Ogi only confirms its adaptive characteristics to a broad range of environments. De Angelis et al [25] also reported that L. rossiae is one of a small number of bacteria that produces Vitamin B12, an essential vitamin needed for human health and well-being. Weissella paramesenteroides has been reported isolated from cucumber with production of bacteriocin [26]. Multidrug resistant gastrointestinal pathogens are increasing. The pathogens used in this study were resistant to Lincomycin, Oxacillin, Cloxacillin, cefuroxime and Ceftazidine. Denver et al [27] reported that Amoxicillin and Cloxacillin has no significant activity against Gram-negative pathogens. Reda et al [28] have shown that some pathogenic isolates from the enterobacteriaceae family (Salmonella spp. Shigella spp. and E. coli) are resistant to Cefuroxime, Ampicillin, and Amoxycillin/Clavulanate. However, the pathogens used in this study were sensitive to the LAB metabolites in cell free supernatant. A previous study has shown the antagonistic activities of cell-free supernatants of LAB against four outbreak strains of pathogenic Vibrio cholerae [29].

The viable cells of the LAB also inhibited the growth of pathogenic bacteria. Several studies have shown that pathogens such as enterotoxigenic E. coli, Shigella flexneri, Salmonella Typhimurium and B. cereus are adversely affected when present in traditional fermented foods [30, 31]. In the present study, W. paramesenteroides AFN004, L. fermentum AFN018, and L. plantarum AFN021, which have recently been isolated from uncooked Ogi (effectively inhibited the growth of Salmonella spp., either when inoculated after 8 hours and 24 hours of growth of pathogen or when cultured overnight and then incubated with the pathogens. In contrast, the growth of the LAB was not significantly influenced by the presence of the pathogens. Szala et al [32] also reported in their studies that during the coculture of Salmonella Senftenberg W775 with the tested strains of Lactobacillus, the concentrations of LAB remained constantly at a level of 107-108 while total inactivation of the pathogen was observed in all the tested mixed cultures. W. paramesenteroides had a broad spectrum of activity against the gastrointestinal pathogens Salmonella sp. SS11 used for the study with a decrease of 6-8 log of the pathogens. Previous studies revealed that the purified bacteriocin from W. paramesenteroides isolated from cucumber exhibited a broad inhibitory spectrum against foodborne pathogens and spoilage microorganisms, such as Salmonella Typhimurium, and Vibrio parahaemolyticus. L. fermentum and L. plantarum reduced the concentration of Salmonella sp. by 6 log [25]. It has been reported that L. fermentum isolated from swine and poultry showed antagonistic effect against Gram-negative bacteria such as Escherichia coli, Salmonella spp., Shigella sonnei, and Gram positive bacteria, Staphylococcus aureus [33]. Alakomi et al [34] reported that organic acids produced by LAB are the agents that inhibited the growth of Gram negative bacteria such as E. coli and Salmonella sp. The present study collaborates our earlier reports of good potentials of LAB in inhibiting growth of pathogens [35–39].

## Conclusion

LAB strains are present in high quantities mostly in omidun followed by uncooked Ogi but reduced quantity in cooked Ogi. The LAB possesses antimicrobial properties against gastrointestinal pathogens with immense antagonistic potential against Salmonella spp. This study has shown that L. rossiae and W. paramesenteroides are parts of the community of microorganisms that colonize Ogi that is regularly consumed by Nigerians. Further in vivo investigation is needed to confirm the gut colonizing properties of LAB from Ogi.

### What is known about this topic

- Lactic acid bacteria is involved in ogi fermentation;
- Inoculation of pathogens directly into ogi and its supernatant inhibits the growth of the pathogens;
- Viable lactic acid bacteria in ogi and omidun.

### What this study adds

- The association of *weissella paramesenteroides, L. rossiae, acetobacter pasteurianus and paenibacillus* sp. with *Ogi* production;
- Deceasing quantities of lactic acid bacteria in Omidun, uncooked Ogi and cooked Ogi;
- Great antimicrobial activities of LAB isolated from ogi against salmonella sp. in coculture.

## Competing interests

The author declare no competing interest.
